# Mast cell metabolism in cancer: an underexplored frontier demanding more attention

**DOI:** 10.3389/fimmu.2025.1693954

**Published:** 2025-11-24

**Authors:** Barbara Frossi, Giuseppina Beatrice Scialpi, Silvia Tonon, Elena Jachetti

**Affiliations:** 1 Immunology Section, Department of Medicine, University of Udine, Udine, Italy; 2 Molecular Immunology Unit, Department of Experimental Oncology, Fondazione Istituto di Ricerca e Cura a Carattere Scientifico (IRCCS) Istituto Nazionale dei Tumori di Milano, Milan, Italy

**Keywords:** immunometabolism, mast cells, tumor microenvironment, tumor metabolism, tumor-stroma crosstalk, metabolic interventions

## Abstract

Cancer metabolism is gaining considerable attention. Tumor cells are characterized by a peculiar metabolic state to sustain the continuous demand of energy and metabolites needed for their proliferation and long-term survival. Such metabolic alterations extend beyond cancer cells, affecting multiple components of the tumor microenvironment (TME), including immune cells, stromal cells, and endothelial structures, and are influenced by both local and systemic conditions. Mast cells (MCs) are innate immune cells capable of both pro- and anti- tumorigenic functions and with the potential to modulate the activity of bystander immune cells. Nevertheless, despite their established importance in the TME, the impact of MCs in modulating cancer metabolism remains largely unexplored. This review outlines current findings regarding the metabolic conditions in the TME that modulate MC function, and, vice versa, how MC-derived metabolites can influence tumor progression, acting both on cancer and stromal cells. We focus on four main altered conditions in the TME: glucose metabolism, amino acid availability, lipid composition, and hypoxia. As studies investigating MC metabolism in cancer are limited, we also discuss relevant literature addressing how metabolic stimuli influence MC activity, as well as the effects of MC-derived metabolites on target cells, in non-cancer physiological or pathological conditions, to highlight possible mechanisms that deserve further investigation in cancer settings. Deeper investigation of MC-related metabolic networks in the TME is needed, not only to elucidate their functional modulation in response to current metabolic interventions, but also to explore their potential as therapeutic targets in the context of cancer metabolism.

## 1 Introduction

To sustain growth, survival, proliferation, and long-term maintenance, cancer cells undergo profound metabolic reprogramming. A well-established feature of this altered metabolic state is the increased uptake of glucose and its preferential conversion to lactate, even in the presence of fully functional mitochondria. This phenomenon is referred to as the Warburg Effect (1). Accordingly, acidification and hypoxia characterize the tumor microenvironment (TME), along with significant changes in the metabolism and availability of amino acids and lipids. These changes affect not only cancer cells, but also all the cells of the TME, including immune cells, stromal cells, and vascular structures. Among these, cells of the immune system should represent a crucial element in tumor progression and a potential therapeutic target (2, 3).

Mast cells (MCs), myeloid cells traditionally known for their role in IgE-dependent allergic responses, play a complex and multifactorial role in the immune response against solid tumors, with both beneficial and harmful effects, depending on tumor type, their peri or intra tumor localization, and interaction with TME elements (4). MCs can help to stimulate the anti-tumor response, but also promote tumor growth and spread, and might represent a novel target for cancer therapy (5).

Although MCs are increasingly recognized as modulators of the TME, their specific contribution to metabolic dynamics is poorly understood. In this work, we present current literature and offer perspectives on this emerging topic. Our aim is to provide an overview of how the metabolic status of the TME impact on MCs activation and functions, and, vice versa, how metabolites derived from MCs can orchestrate molecular and cellular dynamics in the TME. We also highlight potential mechanisms inferred from other cell types and experimental settings that deserve to be better investigated in the context of MCs’ impact in cancer.

## 2 Metabolism of tumor cells and of the tumor microenvironment

Mammalian cells typically generate energy from glucose through three metabolic steps: i) glycolysis, ii) the tricarboxylic acid cycle (TCA), and iii) oxidative phosphorylation (OXPHOS). OXPHOS occurs in mitochondria under normal oxygen conditions and efficiently produces ATP. Under conditions of low oxygen levels or increased energy demand, such as during activation, stress, or proliferation, cells shift from OXPHOS toward glycolysis to rapidly generate ATP. This can occur, for example, in muscle tissues during intense physical activity. This process is called anaerobic glycolysis, or fermentation, is less efficient in terms of ATP yield, and generates lactate as a final metabolic product. Importantly, cancer cells often undergo aerobic glycolysis, characterized by increased uptake of glucose and its preferential conversion to lactate, even in the presence of oxygen and fully functional mitochondria. This phenomenon is the above-mentioned Warburg effect (1). Although aerobic glycolysis is less efficient in terms of ATP yield per glucose molecule, it allows the generation of TCA intermediates for anabolic processes that can support the rapid proliferation of tumor cells. Yet, beyond aerobic glycolysis, tumor cells are highly efficient at adapting to and exploiting multiple metabolic pathways to fulfill the continuous demand for energy and biosynthetic precursors required to sustain their growth and long-term maintenance. These include lipid metabolism, amino acid consumption, and nucleotide synthesis. This metabolic adaptation is so critical for cancer progression that it has been recognized as one of the “Hallmarks of Cancer” (6).

Nevertheless, most of these metabolic pathways are shared between tumor cells and other cell types in the TME, which can compete for metabolites and exert reciprocal influence through altered metabolic activity. These effects are more pronounced in immune cell subsets, and contribute to shape immunosuppression in the TME (2). Although they have been extensively reviewed elsewhere (3), we here briefly summarize the main pathways involved, with the aim of providing an overview of axes and metabolites potentially implicated also in MC function and recruitment.

The main conditions that can influence metabolism and cell function in the TME are: i) glucose metabolism, ii) amino acids availability, iii) lipid composition, and iv) hypoxia. In general, the altered metabolic state characteristic of the TME shapes the function and the metabolism of infiltrating immune cells, towards the development of an immunosuppressive environment, as described below.

### 2.1 Glucose metabolism

As previously mentioned, glycolysis is the primary source of energy for tumor cells. The increased demand of glucose to sustain tumor cell growth leads to a competition for glucose with surrounding cells in the TME, including infiltrating immune cells. For example, effector T cells are highly proliferating cells that rely on aerobic glycolysis to sustain their effector function (7). Therefore, the low glucose availability in the TME blocks T cell activity (8). In parallel, also B cell function can be affected. Indeed, low levels of glucose impair plasma cells differentiation and antibody production (9). In contrast, regulatory T cells (Tregs), which rely more on OXPHOS, are less affected by glucose deprivation and can further contribute to the suppression of T cell responses (7). Furthermore, lactate, the main product of aerobic glycolysis, can directly restrain CD8 T cell activity (10, 11) and sustain Treg function. Lactate has also been shown to induce upregulation of PD1 on Tregs (12) and of PD-L1 on tumor-associated macrophages (TAMs) and myeloid-derived suppressor cells (MDSCs) (13, 14), and also to skew M2 polarization of macrophages (15, 16), thereby further contributing to immunosuppression in the TME.

Besides being influenced by the altered metabolic state induced by the tumor, stromal cells in the TME can also modulate the metabolic behavior of tumor cells. The term “reverse Warburg effect” (17) describes a phenomenon in which stromal cells, mainly cancer-associated fibroblasts (CAFs), undergo aerobic glycolysis, generating high levels of lactate, pyruvate, and ketone bodies. These metabolites can then be taken up by tumor cells, to fuel energy production via OXPHOS, highlighting the metabolic coupling and flexibility within the TME (18). CAFs can also influence metabolic pathways other than glycolysis and OXPHOS in tumor cells. For example, CAF-derived lactate can promote lipid metabolism and epigenetic rewiring in prostate cancer cells (19).

### 2.2 Amino acid availability

To sustain their rapid growth and survival, tumor cells heavily uptake amino acids from the TME, thus reducing their availability for surrounding cells. In particular, tumor cells compete with T cells for key amino acids essential for T cell metabolism and function, including glutamine, arginine, asparagine, leucine, and methionine. Local deprivation of these amino acids in the TME can thus result in impaired T cell activity and immunosuppression (20). Amino acids have also been shown to influence B cell function. In colorectal cancer, a distinct population of immunosuppressive regulatory B cells has been observed to preferentially utilize leucine over glucose as a metabolic substrate. This metabolic preference suggests that leucine deprivation may disrupt the immuno-evasive mechanisms employed by the tumor (21).

Besides this nutrient competition, tumor cells and other immunosuppressive populations in the TME can convert amino acids into metabolites that inhibit T cell function. This is the case of arginine, which is converted into ornithine and urea by the enzymes arginase 1 (ARG1) and arginase 2 (ARG2), expressed by several cancer cells, as well as by MDSCs and TAMs. Arginine depletion compromises T cell functions (22), and ornithine directly suppresses cytotoxic T cell activation (23). Similarly, the enzyme indoleamine 2,3-dioxygenase (IDO) converts tryptophan into kynurenine, a metabolite that promotes T cell exhaustion (24) as well as recruitment and polarization of Tregs, the latter acting via stimulation of the aryl hydrocarbon receptor (AHR) (25).

### 2.3 Lipid composition

As cancer progresses, the lipid composition in the TME undergoes significant changes that affect not only cancer cells, but also neighboring cells (26). CAFs and adipocytes actively secrete fatty acids (27, 28), whereas dying or stressed cells release lipids or lipid vesicles into the TME (29, 30). In this context, cancer cells rewire their lipid metabolism by increasing lipogenesis, lipid uptake, and fatty acid oxidation. This is essential to sustain energy requirements and synthesis of the plasma membrane, as well as to foster certain oncogenic pathways (31). Besides, increased lipid accumulation in the TME can profoundly affect immune cell subpopulations, contributing to both pro-tumor and immunosuppressive effects (3). Lipid uptake in dendritic cells (DCs) induces endoplasmic reticulum stress and impairs antigen presentation capability (32). Moreover, lipid accumulation in macrophages favors their M2 polarization (33), which has pro-tumoral and immunosuppressive functions. Furthermore, different populations of lipid-loaded macrophages have been described to promote tumor growth, as well as invasiveness and mesenchymal differentiation, in prostate cancer (34) and glioblastoma (35), respectively.

Lipid metabolism also plays a crucial role in T cell function within the TME, although the evidence collected so far remains contradictory. On the one hand, CD8^+^ T cells can increase lipid uptake and fatty acid catabolism in the effort to adapt to the hostile metabolic environment and preserve their functional activity within the TME (36). On the other hand, increased lipid, and in particular cholesterol, uptake mediated by CD36 can contribute to ferroptosis and dysfunction of CD8^+^ T cells (37). Furthermore, in pancreatic cancer, it has been shown that the accumulation of long-chain fatty acids drives mitochondrial, metabolic, and functional impairment in intratumor CD8^+^ T cells (38). However, these negative effects are not shared across all long-chain fatty acids. Indeed, linoleic acid has been reported to sustain CD8^+^ T cell function in the TME (39).

Furthermore, short-chain fatty acids (SCFAs), metabolic products of gut bacteria, play a role in tumor pathogenesis. SCFAs themselves or SCFA-producing bacteria are found to be decreased not only in colorectal cancer (40), but also in many other tumor types, including, for example, prostate (41) and lung cancer (42). Alterations in SCFAs can impact on cancer onset and progression. This is because SCFAs are able to influence the TME by altering gene expression through multiple mechanisms, including epigenetic modification of tumor cells themselves and of bystander immune cells (43), but also by influencing the response to immunotherapy (42). For example, the SCFAs pentanoate and butyrate can modulate the anti-tumor immune response by enhancing the production of TNFα and IFNγ by cytotoxic CD8 T cells, thus enhancing their anti-tumor activity (44).

Finally, although lipid metabolism is often associated with impaired activity of effector T cells, it is essential for proper suppressive function and overall fitness of Treg (45). This is achieved through both uptake of extracellular lipids (46) and *de novo* fatty acid synthesis (47).

### 2.4 Hypoxia

Hypoxia (low oxygen levels) is a defining feature of many solid tumors and can profoundly influence the phenotype and function of several cell types in the TME, mainly promoting an immunosuppressive environment (48). For example, it is widely established that under hypoxic conditions TAMs are polarized towards M2 phenotype (49–52). Nevertheless, recent data suggest that hypoxia can also promote immunogenic properties of macrophages, boosting T cell-mediated responses in the TME (53). Hypoxia also promotes the expression of immunocheckpoint molecules such as PD-L1 and VISTA on both tumor cells and MDSCs (54, 55), as well as other suppressive markers (LAG3, TIM3, and CTLA4) on T cells (56), which rapidly undergo exhaustion in the hypoxic TME (57). Also, hypoxia fosters IL-10 expression in B cells, pushing them towards an immunosuppressive phenotype (58). Hypoxia can further contribute to immunosuppression by promoting the release of soluble factors (e.g. TGFβ, IL-6, IL-10, VEGF) by CAFs (59).

As outlined above, metabolic conditions have a profound impact on immune cells populating the TME. Indeed, altered glucose metabolism, amino acids availability, lipid composition, and hypoxia mainly contribute to immunosuppression, although some particular lipids have been shown to promote T cell function. This chapter discussed these metabolic conditions individually. However, it is important to highlight that the metabolic pathways within tumor cells and the TME are complex and highly interconnected, and can be influenced by several factors acting at both local and systemic level, including tumor type and localization [e.g. primary or metastatic (60)], diet (61), physical activity (62), and nutritional and metabolic status of the patients (60, 63). Local tumor heterogeneity and differences in TME composition can further influence cell metabolism (64). Notably, the gut and tumor microbiota can also regulate metabolic pathways and serve as sources of metabolites at the systemic and TME levels, respectively (65, 66).

In the following sections, we will focus on MCs: first by introducing their biology and metabolism in physiologic conditions, then by briefly illustrating their roles in cancer, and finally by dissecting what is known about MC metabolism in the TME. Given the limited literature on this topic, we also integrate findings from non-tumor contexts and from metabolites produced by other cell types, also known to be produced by MCs, to speculate on potential metabolic-related MC functions in the TME that warrant future investigation.

## 3 Mast cell biology

MCs are innate immune cells of the myeloid lineage widely distributed throughout mucosal and epithelial tissues of the body, and most abundant in tissues that serve as barriers to the external environment, such as the gastrointestinal tract, the skin, and the respiratory epithelium (67–69). At these sites, MCs act as “immune sentinels” that can rapidly sense and respond to environmental changes, exerting either protective or detrimental roles depending on the context, in different pathologic conditions such as both acute and chronic inflammation, infection, allergy, autoimmunity, and cancer immunity (70, 71).

MCs can originate from both embryonic yolk sac progenitors and bone marrow-derived hematopoietic stem cells. Yolk sac-derived MCs populate fetal tissues before bone marrow hematopoiesis begins and persist into adulthood, especially in connective tissues like skin and adipose tissue. As development progresses, MCs originate from CD34^+^ progenitors that leave the bone marrow, circulate in the blood, and home to tissues. There, they mature under the influence of the stem cell factor (SCF), and complete their differentiation process by acquiring a specific phenotype depending on local microenvironment signals (72).

Since their discovery, human MCs have been classified in subtypes based on the content of their cytoplasmic granules (73): MC_T_ expressing tryptase only, predominantly located in the respiratory and intestinal mucosa, where they colocalize with T lymphocytes; MC_C_, exhibiting chymase without tryptase, predominantly observed in the submucosa and mucosa of the stomach, small intestinal submucosa, and colonic mucosa (74); MC_TC_, which contain both tryptase and chymase, along with other proteases such as carboxypeptidase A and cathepsin G (73), and are predominantly present in connective tissue areas, such as the skin, submucosa of the stomach and intestine, breast parenchyma, myocardium, lymph nodes, conjunctiva, and synovium (75).

However, recent studies have shown that the traditional classification of MCs is overly simplistic and overlooks their diversity. In 2023, Tauber and colleagues identified six distinct MCs clusters through transcriptomic profiling across 12 human tissues (68). These clusters, defined by specific gene sets, are distributed in various organs. Nevertheless, there were organ-specific enrichments per cluster, suggesting that different MCs found in the same tissue could share the expression of common genes. This heterogeneity goes well beyond the traditional classification of MCs based on protease contents, showing how these cells specialize depending on the tissue in which they reside (68).

The diversity of MCs also emerges from the expression of a wide array of surface receptors, which are essential for the identification of invading pathogens and for the reaction to different stimuli present in the microenvironment. These receptors include the high-affinity receptor for IgE (FcϵRI) and the low-affinity receptor for IgG (FcgRII), the TLRs, the G-coupled receptor MGPRs [e.g. MRGPRX2 (76)], and several receptors for cytokines, neuropeptides and adhesion molecules (77). Moreover, MCs are capable of synthesizing, storing, and secreting an extensive assortment of molecules classified into small-molecule mediators (e.g. histamine, serotonin), protein mediators (e.g. cytokines, proteases), lipid mediators (e.g. leukotrienes, prostaglandins), and proteoglycans (e.g. heparin). Some of the mediators are stored in granules (histamine, proteases, proteoglycans, and small amounts of TNFα) and therefore can be released within seconds or minutes. Others can be newly synthesized within minutes to hours upon stimulation of the cells (e.g. lipid mediators and most cytokines) and often require *de novo* transcription (78, 79). This enormous array of mediators further explains how MCs can be involved in so many different physiologic and pathophysiologic functions (71). Indeed, by acting both as sentinels and as potent modulators of the microenvironment, MCs can integrate environmental and microenvironmental signals and transmit this information to the adaptive arm of the immune system, thereby influencing the development of a polarized immune response (71). In addition, MCs aptitude to calibrate their response depending on which stimulus they receive indicates that MCs can regulate not only the onset but also the amplification, the extension, and the resolution of the immune response.

## 4 Mast cell metabolism

Although there are a few reports describing different non-secretory MC functions (for example phagocytosis), the biological activities of MCs are mainly associated with their unique ability to store and release biologically active compounds (80), also undergoing multiple cycles of degranulation. To fully regenerate the repertoire of granules populating their cytosol, significant protein and lipid synthesis need to occur in concert with intracellular vesicle trafficking and packaging of various components into the granule structure (81). These activities require significant energy, including the employment of classical metabolic pathways such as glycolysis, OXPHOS, and fatty acid oxidation (FAO), but are also dependent on amino acid and lipid availability, and force MCs to undergo continuous metabolic reprogramming.

**Figure 1:**
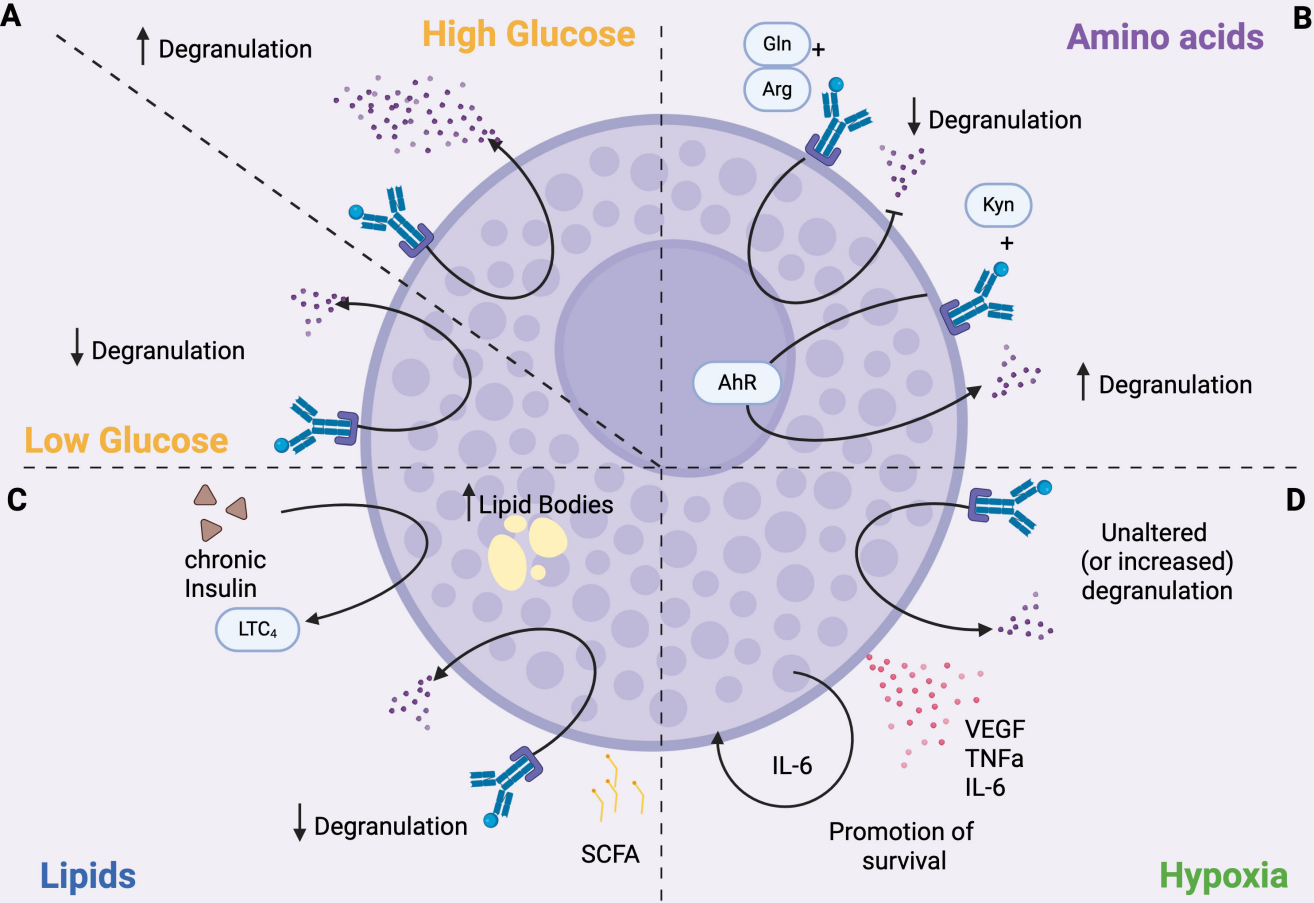
MC function is influenced by metabolism. **(A)** Glucose. Degranulation is enhanced under high glucose conditions and inhibited when glucose levels are low. **(B)** Amino acids. Degranulation is modulated by amino acids; for example, glutamine (Gln) and arginine (Arg) inhibit degranulation, whereas kynurenine (Kyn) enhances it by activating the aryl hydrocarbon receptor (AhR). **(C)** Lipids. SCFAs inhibit degranulation. Chronic insulin exposure increases lipid body formation in MCs and promotes the production of LTC_4_. **(D)** Hypoxia. MCs can survive in hypoxic environments. Hypoxic conditions do not affect, and sometimes increase, degranulation, but induce the secretion of pro-inflammatory cytokines, such as IL6 that promotes MC survival.

The majority of research on MC-metabolism derives from papers published mainly in the early 2000s, and some of them even decades before. Most studies have been conducted on MC lines and murine bone marrow-derived MCs (BMMCs), and in the context of FcεRI-mediated activation. Nevertheless, we can deduce certain considerations from alternative activation pathways. Evidence on how the main metabolic conditions can influence MC activation is reported below (**Figure 1**).

### 4.1 Glucose metabolism

In the 1960s, Chakravarty described a positive correlation between histamine release and increased glycolytic rates in *ex vivo*-isolated rat MCs (82), while, just 10 years later, other groups demonstrated that antigen challenge caused a reduction in pyruvate (83) and ATP levels (84) in rat MCs as a consequence of increased glucose metabolism. Indeed, Chakravarty also observed that the inhibition of glucose uptake obtained with 2-deoxy-glucose (2-DG) (85, 86) or the depletion of glucose from the culture medium (87) reduced histamine release upon antigen stimulation of IgE-pre-sensitized MCs, supporting this evidence. It was suggested that this could be due to the inhibition of ATP-dependent calcium mobilization from intracellular calcium stores. Interestingly in a glucose-free oxygenated medium, 2-DG reduced histamine release induced by antigen stimulation, but not that induced by compound 48/80 in rat MCs (86). When MCs were stimulated in the presence of cyanide, which blocks OXPHOS and is known to blunt both antigen-dependent and compound 48/80-stimulated histamine release, 2-DG potentiated cyanide-induced inhibition of both pathways (86). Glucose restored histamine release under these conditions, but this recovery was completely prevented when 2-DG was added (86). Also, Caslin and colleagues demonstrated that stimulation of BMMCs with IL-33 significantly increased glycolysis and OXPHOS, resulting in the production of pro-inflammatory cytokines, IL-6, TNFαα and MCP-1. Inhibition of OXPHOS had little effect on cytokine production, but antagonizing glycolysis with 2-DG suppressed IL-33 signaling (88). These data support the notion that different mechanisms and different metabolic pathways could be involved in IgE-dependent and independent MC activation.

Experiments conducted with the rat basophil leukemia cell line RBL-2H3 revealed that FcεRI-mediated activation resulted in reduced activity of the glycolytic enzyme M2-type pyruvate kinase (PKM2), which regulates the terminal step of glycolysis (89), likely inducing the accumulation of glycolytic intermediates within MCs. Interestingly, FcεRI-mediated inactivation of PKM2 was shown to be required for MC degranulation *in vitro*, confirming a link between MC metabolism and effector function (89).

In line with the previously outlined glucose-dependency of MC degranulation, it has been demonstrated that seven days of culture of BMMCs with high glucose concentration results in an augmented FcεRI-dependent release of β-hexosaminidase and leukotriene C4 (LTC_4_) without significant alterations in terms of intracellular ATP levels, calcium signaling, or IL-6 secretion (90). Mechanistically, such culture conditions induced a FcεRI-dependent phosphorylation at residue Ser505 of the cytosolic phospholipase A2 that contributes to the enhancement of LTC_4_ secretion (90). This is particularly interesting because MCs cultured in the presence of high levels of glucose showed increased degranulation even upon stimulation with suboptimal antigen concentrations, meaning that prolonged exposure to high glucose may increase MCs’ sensitivity to low antigen doses, potentially lowering the threshold for triggering allergic reactions. Indeed, these results indicate that glucose availability acts as a regulating factor for FcεRI-mediated MC responses (**Figure 1A**). Similarly, the culture of the human MC lines HMC-1 and LAD2 in high-glucose medium increased the levels of intracellular ROS and the phosphorylation of several members of the MAPK family (ERK, JNK, and p38), which in turn promoted the production of pro-inflammatory cytokines (TNFα, IL-1β, IL-6) and of Th2 cytokines such as IL-13 (91). Of note, β-hexosaminidase production was increased in LAD2 cells, while its release was independent of glucose concentration (91).

More recently, by performing Seahorse assays on BMMCs, Phong and colleagues showed a rapid and robust increase in glycolysis, measured as extracellular acidification rate (ECAR), that peaked within ten minutes and persisted for over two hours after MC stimulation with IgE-antigen (92). This response was closely associated with antigen concentration and FcεRI binding affinity: an antigen with high valency stimulated an immediate increase in glycolysis, whereas a low valency antigen did not (92). Interestingly, a slight increase in ECAR was already observed in MCs sensitized with IgE in the absence of antigen (92). These data suggest that antigen concentration influence glycolytic response upon IgE stimulation, and may help to explain the differences reported in the aforementioned studies.

OXPHOS also contributes to IgE-antigen-mediated degranulation of primary human and mouse MCs *in vitro*, in a MAPK- and STAT3- mediated manner (93). This suggests that not only glycolysis but also mitochondrial ATP production can fulfill the energy demands of activated MCs, and highlights MAPK signaling as a key pathway linking MC metabolism to their functional response. MC degranulation can also occur in glucose-free medium, *in vitro* (93). Interestingly, the aforementioned paper of Phong (92) also described that antigen cross-linking did not immediately alter mitochondrial respiration, as the oxygen consumption rate (OCR) was relatively unchanged despite a small decrease soon after FcεRI engagement. However, OXPHOS was required for late-phase responses and for both MC degranulation and cytokine production (92). The observed time-dependent differences in MC mitochondrial respiration were suggested to result from complex transcriptional reprogramming events, which have longer lead times and are therefore not observed in acute MC stimulations (92). Of note, the same study showed that FAO is dispensable for MC activation *in vitro*, since the FAO inhibitor etomoxir neither inhibited IgE/antigen-induced MC degranulation nor IL-6 production (92).

Recent findings have further clarified that glucose metabolism is crucial in influencing MC reactivity *in vivo* (94). In a streptozotocin-induced mouse model of high-glucose diabetic milieu, Yao and colleagues demonstrated that glucose uptake lead to ERK1/2 phosphorylation in MCs (94). Also, prolonged glucose stimulation triggers mTOR hyperactivation, leading to endoplasmic reticulum and mitochondrial oxidative stress, which in turn blunt mitochondrial functions of MCs. Consequently, MCs degranulate and release histamine, tryptase, and inflammatory factors into the neural microenvironment contributing to neuropathy in diabetic mice (94). Furthermore, chronic insulin exposure induces a steatotic phenotype in MCs, characterized by the accumulation of lipid bodies. This state is associated with reduced histamine release but enhanced production of bioactive lipid mediators, with significant alterations in lipid classes involved in the inflammatory response (95). Functionally, FcϵRI-mediated activation under insulin exposure affects the release of LTC4, prostaglandin D_2_ (PGD_2_), and resolvins, suggesting a direct impact of hyperinsulinemia on the regulation of both pro-inflammatory and pro-resolving pathways (95).This evidence suggests that glucose availability acts as a regulating factor for MC responses, where glycolysis is essential for MC immediate degranulation, while mitochondrial respiration is employed in later responses.

### 4.2 Amino acid availability

MCs utilize a complex amino acid metabolism for their various functions, including histamine synthesis, protease production, and energy generation. Amino acids are essential for MCs to build peptides and proteases, like tryptase and chymase. These molecules constitute the MC granule repertoire at steady state that is continuously replenished after emptying (96).

Notably, histamine is derived from the amino acid histidine through the enzyme histidine decarboxylase (HDC) within the Golgi apparatus and stored in granules (97). Histidine deprivation could impair MC development as histamine itself is needed for their full maturation. Furthermore, in HDC-deficient mice that are unable to synthesize histamine, peritoneal MCs showed poorly formed secretory granules, containing lower levels of protease (98). Exogenous histamine partially restored granule differentiation, as shown by increased tryptase and chymase activity, in a manner dependent on histamine receptor type H4. However, H4-deficient mice exhibited normal granule formation in peritoneal MCs, suggesting that endogenous histamine is sufficient for most granule maturation processes when HDC is functional, rendering H4 dispensable (98).

Several *in vitro* studies demonstrated that glutamine and arginine exert anti-inflammatory effects by decreasing the release of *de novo* synthesized leukotrienes and cytokines after IgE-dependent MC activation (99) (**Figure 1B**). Conversely, a link between glutamine and intestinal MC activation in the process of fat absorption was demonstrated *in vivo* (100). Indeed, it has been observed that the absorption of triglycerides as well as the levels of mucosal MCs protease II, histamine, and prostaglandin D2 (PGD_2_) are increased in the circulation following the enteral administration of L-glutamine in rats fed with a lipid meal (100). Thus, L-glutamine could specifically activate MCs to degranulate during fat absorption. We think that this observation has potentially clinical relevance since L-glutamine is often used to promote gut health and repair leaky gut.

Similarly, tryptophan metabolism also influences MCs responses. The tryptophan-derived metabolite kynurenine, but not kynurenic acid and quinolinic acid, has been demonstrated to increase IgE-mediated MC responses through AHR signaling both in mouse and human MCs (101), as shown in **Figure 1B**. Indeed, kynurenine promotes MC degranulation, lipid metabolite production, and IL-13 secretion through activation of PLCγ1, Akt, and MAPK p38, and enhancement of calcium signaling mediated by AHR engagement. Notably, different MC responses can be achieved depending on the duration of AHR stimulation: *in vitro*, a single dose boosted proinflammatory features such as histamine and IL-6 release, whereas continuous stimulation shifted MCs toward impaired degranulation and IL-17 production (102). This let us to hypothesize that changes in the metabolism resulting in increased production of AHR ligands could affect MC functions.

### 4.3 Lipid composition

MCs undergo a dramatic membrane reorganization during degranulation and granule recovery that implies deep changes in their lipid profile. Indeed, MCs generate a variety of bioactive lipids, including leukotrienes, prostaglandins, sphingolipid metabolites, and platelet-activating factor (PAF), which contribute to the fine-tuning of allergic responses by regulating the functions of various cell types (103, 104). Moreover, MC reactivity is modulated by lipid mediators produced both by neighboring cells exposed to environmental challenges and by MCs themselves in an autocrine manner (105).

Molecules like lysophosphatidylinositol (LPI), lysophosphatidic acid, sphingosine-1-phosphate (S1P), prostaglandins, and leukotrienes can activate MCs, leading to chemotaxis, cytokine synthesis, and changes in cytoskeletal dynamics (103). For example, LPI induces strong MC recruitment and cytokine production, with different receptors mediating each response (106).

On the contrary, other lipid mediators can reduce the reactivity of MCs. For example, endocannabinoids, bioactive lipids serving as secondary immune modulators, have been demonstrated to down-regulate MC-mediated inflammatory processes. Indeed, MCs constitutively express the type-1 (CB1) and type-2 (CB2) G protein-coupled cannabinoid receptors, whose engagement by anandamide restrains MC degranulation and cytokine synthesis (107). SFCAs also show different effects on MC response, predominantly inhibiting MC function through butyrate and propionate but not acetate (108).

Several studies have also shown the importance of lipid metabolism in MCs that goes beyond the production of lipid mediators. As previously described, chronic insulin exposure is associated with elevated lipid body numbers, overall increase in cellular lipid content, and elevated LTC4 production (**Figure 1C**) in both cell model (RBL2H3) and primary MCs (95, 109). Also, cholesterol and high-fat diet increased MC degranulation and circulating histamine levels in mice (110). Interestingly, high fat diet also increased the number of MCs in the arcuate nucleus of the hypothalamus in mice (111). There, MCs activate microglia, which in turn suppresses the activity of proopiomelanocortin neurons, increasing appetite and reducing energy expenditure, thus leading to obesity (111). Notably, leptin deficiency impairs MC signaling and alters the balance between pro- and anti-inflammatory cytokines, preventing the development of obesity in mice (112).

So, different reactivity to lipid activating signals, changes in local lipid composition induced by stimuli, and alterations in lipid transport can modulate MC responsiveness (103). Although lipidomic data on MCs appear to be still incomplete, the possibility of modulating their reactivity through lipids is rapidly emerging as a new way to target and control MC responses.

### 4.4 Hypoxia

Hypoxia is a common state in tumors as well as in inflamed tissues, and influences the behavior of MCs, which are highly sensitive to changes in oxygen levels. *In vitro* experiments showed that human cord blood-derived MCs can survive hypoxia, an effect sustained by autocrine production of IL-6 triggered by hypoxic conditions. The same study also demonstrated that hypoxia does not alter MC degranulation, although it can inhibit specific cytokine production after LPS or CD30 treatment (113) (**Figure 1D**). Yet, another study showed that systemic hypoxia can trigger MC degranulation, which can be prevented by treatment with lipoic acid and nitric oxide (114). Moreover, inhibition of MC degranulation with cromolyn prevented or reduced the hypoxia-induced increase in ROS production, leukocyte adhesion and migration, as well as vascular permeability, in a rat model of systemic hypoxia (114). As shown in **Figure 1D**, hypoxia also triggers VEGF production by BMMCs, via the activation of Fyn kinase (115), as well as their secretion of proinflammatory cytokines, including TNFα and IL-6 (116).

To sum up, it is evident that glucose, lipid, and amino acid metabolism undergo profound changes during MC activation and response (**Figure 1**). Variations in the availability of these metabolites can increase or reduce MC ability to degranulate and produce cytokines, thereby modulating their contribution to the immune response.

## 5 Mast cell functions in cancer

MCs have increasingly emerged as key players in the TME, yet, their functions remain context-dependent and controversial, as they may either facilitate or restrain tumor progression under different conditions. Here, we briefly outline the potential roles of MCs in the TME, which have been extensively reviewed elsewhere (117–122). This chapter provides the necessary context for the subsequent discussion of metabolic-related functions of MCs in cancer, while referring readers to the existing literature for a comprehensive overview.

MCs can release proangiogenic factors such as VEGF, FGF-2, PDGF, and proteases like tryptase and chymase, which support tumor growth and metastasis. They also secrete matrix metalloproteinases, mainly MMP9, to further support invasion and metastasis (119). These functions have been documented in several types of cancers, including pancreatic, thyroid, bladder, and colon cancer (123–126).

Conversely, MCs also display antitumorigenic properties, which have been mainly associated to their production of IL-6 in melanoma and lung cancer (127), and of TNFα in neuroendocrine prostate cancer (128). Notably, a pan-cancer analysis showed that MCs correlate with good prognosis in nasopharyngeal cancer due to high TNFα production and a favorable TNFα/VEGF ratio, whereas TNFα-negative, VEGF-producing, MCs associate with poor outcomes in lung, colon, pancreas, and kidney cancers (129).

Furthermore, the activity of MCs can vary markedly across different histological subtypes of the same tumor. For instance, in breast cancer MCs can promote the growth in luminal subtypes while preventing it in the basal ones (130). Also, the apparently contrasting functions of MCs can be explained by different functions depending on their peri- or intra- tumor localization, as shown in prostate cancer (131, 132).

Finally, MCs can actively interact with other immune cells in the TME, thereby contributing to the regulation of either immunosuppression or anti-tumor immunity. For instance, MCs foster the recruitment and suppressive function of MDSCs (133–135). Furthermore, MCs promote immunosuppression by secreting adenosine, which hampers NK and effector T cells, also fostering Tregs activity (136). MCs were also shown to drive resistance to anti-PD-1 therapy in mouse models (137). The relationship between MCs and Tregs is indeed well studied and involves reciprocal interactions leading to Treg-Th17 switch (138, 139), and impairment of MC degranulation (140). Conversely, MCs have also been described to support the activity and recruitment of T and NK cells (141, 142), and these effects might have a role in cancer immunity.

Therefore, MCs can exert pleiotropic roles in cancer. To what extent these functions are driven by metabolic underpinnings remains an underdeveloped topic. In the next chapter, we outline what is already known about MC metabolism in the TME, and we speculate on possible new functions that need to be investigated in this context.

## 6 Mast cell metabolism in the TME

**Figure 2:**
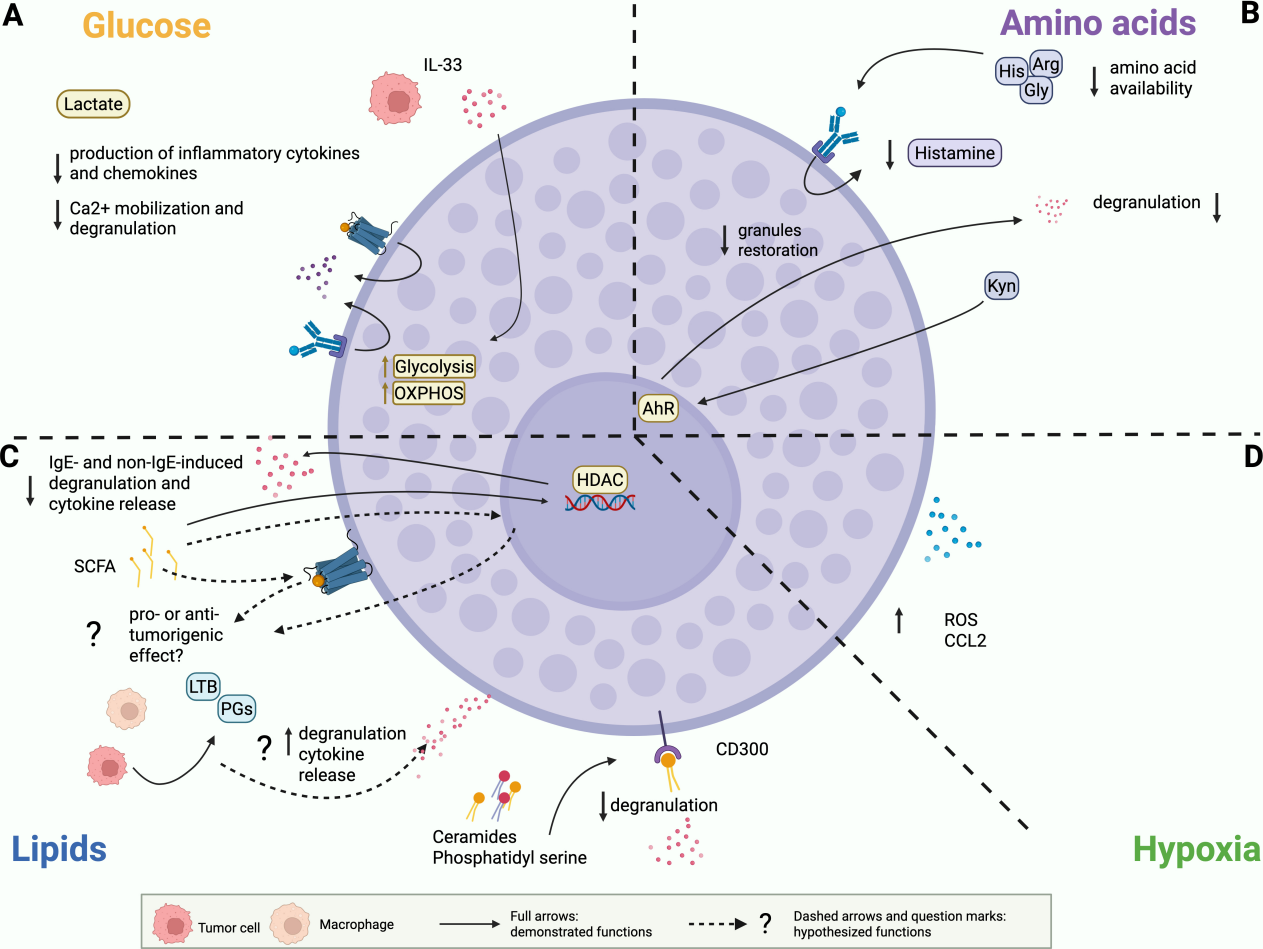
Demonstrated and potential effects of the metabolic environment on MCs. **(A)** Glucose. Lactate inhibits MC degranulation and production of cytokines and chemokines. Tumor-derived IL33 can promote glycolysis and OXPHOS in MCs. **(B)** Amino acids. Reduced availability of glycine (Gly), histidine (His) and arginine (Arg) can reduce MC ability to produce histamine and to restore granule content. Continuous stimulation of AHR by kynurenine (Kyn) can reduce MC degranulation and rewire cytokine release. **(C)** Lipids. SCFAs suppress MC degranulation and cytokine release. Pro or anti-tumor outcomes of these alterations have to be demonstrated. Ceramides and phosphatidyl serine signal on CD300 receptors to inhibit FcεRI mediated MC degranulation. Prostaglandins (PG) and leukotrienes (LTB) can stimulate MC degranulation, cytokine release and migration. This function has been extensively described in allergic contexts and has to be investigated in tumor-infiltrating MC. **(D)** Hypoxia. MCs can accumulate in tumor hypoxic regions, where they secrete reactive oxygen species (ROS) and CCL2.

**Figure 3:**
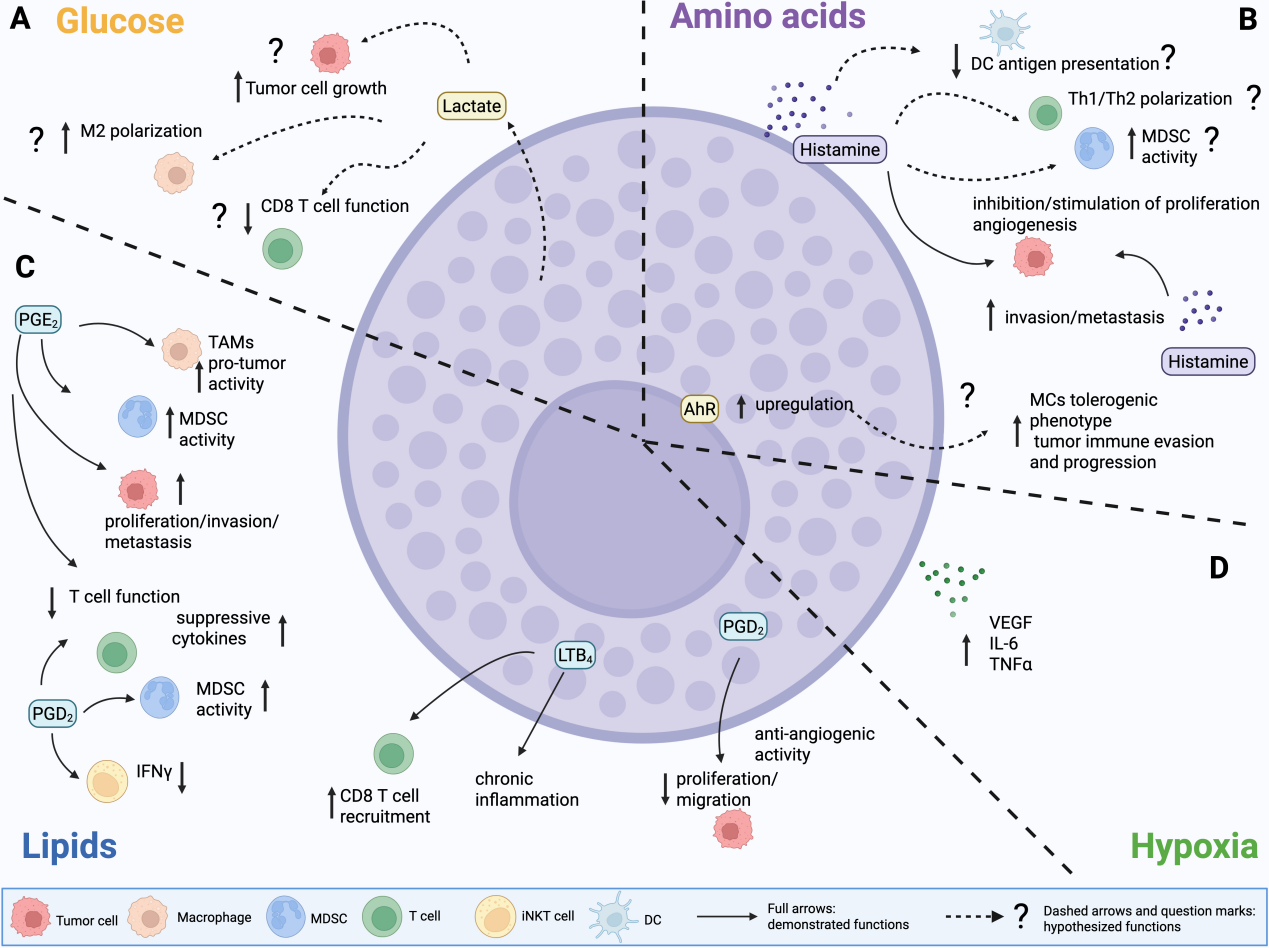
Demonstrated and potential effects of MC-derived mediators and metabolites on the TME. **(A)** Glucose. MCs might release lactate to fuel tumor cell growth and boost immunosuppression. **(B)** Amino acids. MC-derived histamine can either promote or inhibit tumor growth. Histamine produced by other cell types in the TME can also foster invasion, metastasis and angiogenesis, inhibit the function of DCs, foster the suppressive activity of MDSCs and Tregs, and shape Th17/Th2 polarization. Yet, these functions have not been directly linked to MC-derived histamine. **(C)** Lipids. Prostaglandin D_2_ (PGD_2_) produced by MCs can suppress tumor growth and inhibit angiogenesis. PGD_2_ by other cell sources has been also associated to several immunosuppressive functions, including stimulation of TAMs and MDSCs, inhibition of T cell function and reshape towards production of suppressive cytokines, inhibition of IFNγ production by iNKT cells. PGE2 displays similar pleiotropic effects in the TME, however demonstration of PGE2 production by MCs is lacking. Regarding leukotrienes, MC-derived LTB_4_ is associated to chronic inflammation that leads to lung cancer. **(D)** Hypoxia. MCs can release VEGF, IL6 and TNFα, which can exacerbate hypoxia-associated features in the tumor microenvironment (TME).

As summarized in the previous chapter, MCs and their mediators can play context-dependent roles in cancer, influenced by tumor stage, localization, and interactions within other cells (118). Yet, the contribution of MCs in shaping metabolism within the TME is still underexplored, and data on changes in MC metabolism that could occur in tumoral settings are lacking. In this section, we examine the available literature and discuss potential mechanisms through which MCs may influence tumor metabolism and vice versa, distinguishing between (a) effects of TME-derived metabolites on MC activation and recruitment (**Figure 2**), and (b) effects of MC-derived metabolites on cells of the TME (**Figure 3**, **Table 1**).

**Table 1:** Studies that provide direct proof of effect of MC-derived metabolites in cancer context, *in vitro* and *in vivo*.

Mast cell type/ Mouse model	Metabolite	Tumor model	Mechanism	Effect	Reference
*In vitro*					
RBL-2H3 (rat mast cell line)	Histamine	EL4 (murine T lymphoma cell line)	Modulation of histamine receptor H1R, H2R, and H4R on cancer cell	Promotion of EL4 growth	Paudel et al., 2019 Ref. 174
		YAC-1 (murine lymphoblast cell line)	Modulation of histamine receptor H2R, and H4R on cancer cell	Inhibition of YAC-1 growth	
		L1210 (murine lymphocytic B cell line)	Modulation of histamine receptor H1R on cancer cell	No effect	
Cord-blood derived mast cells	Histamine	A549 and LLC (human and murine non-small cell lung cancer cell lines)	Stimulation of the ERK pathway	Promotion of tumor cell proliferation	Stoyanov et al., 2012 Ref. 175
*In vivo*					
Kit^W-sh/W-sh^ mast cell-deficient mice or mice treated with nedocromil sodium	Unknown	Subcutaneous injection of LLC (mouse Lewis lung carcinoma cell line)	Degranulation	Mast cells limit tumor growth	Stoyanov et al., 2012 Ref. 175
Tumor bearing mice treated with cromolyn sodium	Histamine (and possibly other mediators)?	Subcutaneous injection of Mz-ChA-1 (cholangiocarcinoma cell line)	Cromolyn treatment reduces VEGF-C production in the TME	Cromolyn treatment limits tumor growth	Johnson et al. 2018 Ref.176
Kit^W-sh/W-sh^ mast cell deficient mice treated with AOM/DSS reconstituted with wt or H-PGDS^-/-^ MC	PGD_2_	Chronic enterocolitis-associated colon cancer	Inhibition of TNFα production	Mast cells-derived PGD_2_ protects from colitis and colon cancer formation	Iwanaga et al., 2014, Ref. 193
Ki^W-sh/W-sh^ mast cell deficient mice injected with LLC and reconstituted with WT, H-PGDS^-/-^, H-PGDS^-/-^ TNFα^-/-^or TNFα ^-/-^ MC	PGD_2_	Subcutaneous injection of LLC (mouse Lewis lung carcinoma cell line) or B16 (mouse melanoma cell line)	Inhibition of TNF, IL-6, MCP-1, VEGFa production	Mast cells-derived PGD_2_ reduces tumor growth	Murata et al., 2011 Ref. 194
ACKR2^–/–^ Kit^W-sh/W-sh^ Apc^Min/+^ mice	LTB_4_	Apc^Min/+^ spontaneous intestinal adenomas	Recruitment of CD8 cells through BLT1 receptor	Mast cells-derived LTB4 is required for CD8+ T lymphocyte recruitment in the TME	Boddulari et al., 2018 Ref. 208

### 6.1 Effect of TME-derived metabolites on MC activation and recruitment

#### 6.1.1 Glucose metabolism

In the TME, glucose levels are typically reduced while lactate levels are significantly elevated due to the Warburg effect. High levels of lactate function as a feedback inhibitor, thereby limiting the inflammatory response and consequently engendering an immunosuppressive and tumor-tolerant environment. High intracellular levels of lactic acid reduce glucose uptake and suppress glycolytic ATP production, thereby reducing the energy available for signal transmission and cytokine synthesis within the cell. In MCs, increased levels of lactic acid have been demonstrated to exert suppressive effects in both *in vitro* and *in vivo* contexts (**Figure 2A**).

*In vitro*, lactic acid significantly inhibited the production of inflammatory cytokines and chemokines, such as TNFα, IL-6, IL-13, MCP-1, and MIP-1α, by MCs upon stimulation of FcεRI (143) or MRGPRX2 receptors (144), as well as in response to LPS (145) or IL-33 (146). Interestingly, in IL33-stimulated BMMCs lactic acid reduces secretion of cytokines, but it increases VEGF production (146). This effect could play an important role in the context of tumor angiogenesis and should be further investigated.

*In vivo*, lactic acid injection reduced the hypothermia caused by IgE-antigen challenge in mice undergoing passive systemic anaphylaxis and attenuated the anaphylactic reaction (143). Mechanistically, high levels of lactate reduced the phosphorylation of key proteins involved in the signaling cascades, including SYK, BTK, and ERK in IgE-mediated responses (143) and TAK1, JNK, ERK, and NF-κB in IL-33-mediated responses (146). Furthermore, high levels of lactic acid have been observed to impair the effect on Ca²^+^ mobilization, causing reduced MC responsivity during MRGPRX2-mediated responses (144).

To date, the current literature does not provide any data on the effect of lactate on the behavior of MCs in experimental tumor models *in vivo*. However, evidence from systemic anaphylaxis experiments suggests that, in high lactate contexts such as the TME, the ability of MCs to massively degranulate in response to acute stimulation could be reduced. This led us to hypothesize that MCs can be induced into a tolerant state in the TME, where they presumably do not release high levels of inflammatory mediators as a consequence of FcεRI, MRGPRX2, or ST2 stimulation. However, we can speculate that this does not preclude the possibility that MCs could release low levels of cytokines or other mediators, triggered by other stimuli in the TME.

In a recent paper, we compared the bioenergetic profile of BMMCs co-cultured with colon organoids obtained from either intestinal crypts of healthy mice or from adenomas from the azoxymethane (AOM)/dextran sulfate sodium (DSS) mouse model of colon cancer. The extracellular flux analyzer (Seahorse analysis) revealed that glycolysis and ATP production were significantly higher in MCs maintained in co-culture with tumoral organoids than with healthy ones (147), suggesting that MCs are more metabolically active in the presence of tumoral organoids, likely to sustain their activation. This hypothesis was further confirmed by the upregulation of the surface expression of CD107a and by the release of TNFα by MCs incubated with tumoral organoids, an effect dependent on IL-33 released by tumor organoids (147). Since, as described above and shown in **Figure 2A**, IL-33 can increase glycolysis, glycolytic protein expression, and OXPHOS in MCs *in vitro* (88), we hypothesize that the tumor, through the secretion of various mediators such as IL-33, can exert a substantial influence on MC metabolism, consequently modulating their behavior. It is important to highlight that the co-culture with organoids might represent a model for early-stage tumors, so we hypothesize that the TME could exert different effects on MCs depending on the tumor stage, and on the activating (e.g. IL-33) and inhibiting (e.g. lactate) tumor-derived molecules that can shape MC metabolism and functions. Similarly, the metabolic profile of MCs may be influenced by a number of other elements present in the TME beyond tumor cells, potentially exerting divergent effects. The overall outcome on MCs would be determined by the combined effect of all these signals.

#### 6.1.2 Amino acid availability

In the TME, amino acid availability is profoundly altered compared to normal tissues, due to the intense metabolic demands of tumor cells and the interaction with immune and stromal cells. As previously mentioned, MCs require glycine, histidine, and arginine to produce histamine and proteases that are maintained pre-stored in cytoplasmic granules (**Figure 2B**). Consequently, we can speculate that reduced availability of these amino acids in the TME could reduce MC ability to restore granule content after emptying, thereby affecting their capacity to respond to tumor- or stroma-derived stimuli.

Regarding tryptophan metabolism, as previously stated the kynurenine pathway is usually up-regulated in cancer settings. The continuous stimulation of AHR by kynurenine in MCs has been shown to modulate the inflammatory response by reducing degranulation and modifying the pattern of cytokine release (102). Thus, we hypothesize that long-term stimulation of AHR through kynurenine may confer anergy to MCs and prompt them to polarize towards a tolerogenic phenotype, promoting tumor evasion and progression (**Figure 2B**). However, reliable data concerning this topic are not currently available. Consequently, the claims we made herein are merely speculative and require further validation.

#### 6.1.3 Lipid composition

In 2022, Hanahan suggested that polymorphic variation in microbiomes of the intestine and other organs, as well as the tumor microbiome, may constitute a distinctive enabling characteristic for the acquisition of cancer hallmark capabilities (148). Nowadays, it is widely evident that the microbiota is an important element of the TME and that microbial-derived metabolites, such as SCFAs, may be important modulators, with a dual role in promoting or inhibiting cancer progression (149). Folkerts and colleagues (108) demonstrated that butyrate and propionate potently inhibit both IgE- and non-IgE-induced MC degranulation and inflammatory cytokine production (**Figure 2C**). The study revealed that butyrate acts as a histone deacetylase (HDAC) inhibitor (**Figure 2C**), and this reduces the expression of genes crucial for FcϵRI-mediated signaling, such as BTK, SYK, and LAT (108). Butyrate has also been shown to suppress *in vitro* the proliferation of the mouse mastocytoma P815 cell line (150), as well as the production of IL-6 and TNFα but not the release of β-hexosaminidase from BMMCs activated in an IgE-dependent way (151). In apparent contrast with the previous findings, a recent study also showed that sodium butyrate modifies the granularity of MCs and increases heparin content in a time- and concentration-dependent manner, alongside augmented expression of enzymes involved in heparin biosynthesis (152). How these effects could have a role in the tumor context remains unknown. However, since it is well established that SCFAs modify the reactivity of MCs, we can suppose that changes in the levels of SCFAs present in the TME also influence MC behavior. Whether it will be pro- or anti-tumor remains to be demonstrated.

The CD300 receptor family (153) might also link lipid sensing to MC function in the TME. CD300a and CD300f are inhibitory receptors that recognize structural lipids such as extracellular ceramides and phosphatidylserine (PS). Normally, PS becomes exposed on the plasma membrane during apoptosis and signals phagocytes to trigger efferocytosis, a process commonly dysregulated in cancer progression and immune evasion (154). In MCs, the binding of PS and ceramides to CD300a and CD300f, respectively, inhibits FcϵRI-mediated activation, ultimately suppressing MC degranulation and attenuating inflammation (**Figure 2C**) (155). To date, there are no studies directly addressing whether the triggering of CD300 receptors on MCs contributes to their functions within the TME. However, CD300 family members have been implicated in cancer progression via modulation of other immune cell populations or by directly influencing tumor cells. There is growing evidence suggesting that CD300a is involved in the development of hematological malignancies. In acute myeloid leukemia, the knockdown of CD300a reduced tumor cell proliferation and migration while promoting apoptosis (156). Additionally, the knockdown of this receptor can inhibit cell growth and division in diffuse large B-cell lymphoma cells, but has no impact on cell apoptosis (157). Even though the role of CD300f in cancer has not been dissected so far, it has been shown that the triggering of CD300f upregulates PD-L1 expression in human monocytes and macrophages, thereby fostering their suppression of T cell proliferation (158). Consequently, CD300f blockade may represent a potential therapeutic strategy in cancer treatment. Therefore, we can speculate that even MC-expressed CD300a/CD300f might sense ceramides and PS in the TME, likely contributing to pro-tumorigenic processes. The biological outcome in this context might depend on the local immune landscape and ligand availability, and should also be interpreted in light of the aforementioned inhibitory effect of CD300 engagement on MC degranulation.

Many other lipid mediators play a crucial role in the TME, including prostaglandins, leukotrienes, PAF, and S1P. These bioactive lipids can be released by tumor cells and myeloid cell subsets, as well as by MCs themselves. In the TME, such mediators can contribute to cancer progression by fostering tumor cell proliferation, angiogenesis, metastasis, and immune evasion (159–161). Notably, MCs express receptors for these categories of lipid mediators, which, depending on the context, can induce degranulation, cytokine release, chemotaxis, or prolonged survival, as exhaustively reviewed elsewhere (155, 162). Interestingly, prostaglandin E_2_ (PGE_2_) can either restrain or stimulate MC function and degranulation depending on which receptor is triggered on their surface (163). PGE_2_ is also a potent chemoattractant for MCs (164). Furthermore, *in vitro* experiments with BMMCs and human cord blood-derived MCs showed that leukotriene B4 (LTB_4_) recruits immature MC precursors, suggesting that this leukotriene could regulate MC density in tissues in an autocrine way (165). Even if these functions have been described in the context of allergies and immune-related disorders, we can reasonably hypothesize that similar mechanisms may also occur within the TME, likely influencing the crosstalk between MCs and tumor or stromal partners (**Figure 2C**).

#### 6.1.4 Hypoxia

As previously mentioned, MCs can survive in hypoxia. Indeed, in the murine B16-F1 melanoma model it has been shown that MCs accumulate in hypoxic regions, where they secrete reactive oxygen species (ROS) and CCL2 (**Figure 2D**) (166). Hypoxia can also promote pro-angiogenic functions of MCs by stimulating their release of VEGF (115). We can therefore speculate that MC can contribute to the regulation of tumor development in hypoxic conditions.

In conclusion, findings so far suggest that altered glucose metabolism and amino acid availability in the TME may impair MCs functions. On the contrary, different lipidic products potentially available in the TME can promote or inhibit MC degranulation, and cytokine production (**Figure 2**). Yet, as these data were mainly collected from *in vitro* experiments or from non-cancer settings, it still remains to be elucidated if these effects are actually occurring and relevant in the tumor context*, in vivo*.

### 6.2 Effect of MC-derived metabolites on the TME

#### 6.2.1 Glucose metabolism

As stated above, lactate can profoundly impact MC activation. Nevertheless, MCs themselves have been described to be able to release lactate, in association with histamine (167). Even if this work focused on an allergic context, and a direct proof of lactate production by tumor-infiltrating MCs is still lacking, this evidence suggests to us the possibility that MCs could directly impact glucose metabolism and tumor development by releasing lactate in the TME (**Figure 3A**). This hypothesis warrants further investigation.

#### 6.2.2 Amino acid availability

Regarding amino acid metabolism, as stated histamine, one of the main mediators of MCs, is a biogenic amine, produced starting from the amino acid histidine via the HDC enzyme. Histamine can also be produced by other cell types, including tumor cells themselves, and exerts diverse effects in the TME, impacting cancer progression by both acting directly on tumor cells and modulating immune and stromal elements (168, 169). Histamine can either promote or inhibit tumor growth, depending on which cell type and which of its four receptors (H1R~H4R) is engaged (168). Histamine receptors (HR) H1R, H2R, and H4R are widely expressed by immune cell subsets, and the former also by endothelial and epithelial cells, whereas H3R is mainly expressed by neural cells. All the receptors can be present on tumor cells. Histamine has both stimulating and suppressive functions on immune cells (169): for example (**Figure 3B**), it can both promote or inhibit antigen presentation and function of dendritic cells (170–172), and influence Th1/Th2 polarization (171). It can also prompt immunosuppression by fostering the activity of Treg (173) and MDSCs (174). Furthermore, histamine can directly sustain tumor cell proliferation, invasion, and metastasis, as well as stromal remodeling and angiogenesis (**Figure 3B**) (168). In light of these pleiotropic functions, HR inhibitors are now being investigated as a possible therapeutic tool in cancer (168).

Yet, literature showing a direct effect of MC-derived histamine in the TME is limited (**Figure 3B**). Intriguingly, it was demonstrated that it exerts opposite effects on different T cell lymphoma cell lines. Specifically, *in vitro* experiments with the RBL-2H3 rat mast cell line showed that MCs inhibit the growth of YAC-1 cells, promote the proliferation of EL4 cells, while have no effect on L1210 cells (175). This divergence was linked to MC-mediated modulation of HR in tumor cells. Specifically, it involved downregulation of H2R and H4R in YAC-1 cells, upregulation of H1R, H2R, and H4R in EL4 cells, and modulation of H1R alone in L1210 cells. These changes in HR expression levels resulted in distinct downstream signaling events, affecting cell survival, apoptosis, mitochondrial integrity, and cell cycle regulation in the respective tumor cell lines (175). Notably, MC-derived histamine was also shown to promote the proliferation of human (A549) and murine (LLC) lung adenocarcinoma cells, *in vitro* (176). However, the effect was opposite *in vivo*, where MCs exhibited anti-tumor activity in the mouse LLC model (176). Nevertheless, the direct activity of MC-derived histamine was not investigated *in vivo*, and experiments relied only on injection of tumor cells in Kit^W-sh/W-sh^ MC-deficient mice or in mice treated with nedocromil sodium to block MC degranulation (176). Finally, in a mouse model of cholangiocarcinoma, MC-derived histamine has been proven to support tumor growth, EMT, and angiogenesis. Indeed, the authors demonstrated that blocking the release of histamine by MCs using cromolyn sodium not only resulted in smaller tumor masses in mice but also in decreased expression of VEGF-C, released by human fetal-derived MCs *in vitro*, as well as *in vivo* in the TME (177).

Notably, among the key amino acids that are crucial for both tumor cell growth and T cell activation in the TME (previously described in this review), methionine seems necessary for phospholipid methylation and consequent histamine release by MCs after IgE stimulation (178). Finally, it has been also shown that canine MC tumor cells can express IDO (179). Yet, the contribution of MCs to IDO production, tryptophane deprivation, and related immunosuppression in the TME still needs to be elucidated in human cancer.

#### 6.2.3 Lipid composition

MCs produce a variety of lipid mediators (shown in **Figure 3C**), mainly including derivatives of arachidonic acid. This fatty acid can be metabolized through three different pathways: the cyclooxygenase (COX) pathway, leading to the production of prostaglandins, the lipoxygenase pathway, mediating the synthesis of leukotrienes, and the cytochrome P450 pathway, which produces EET, 19-HETE, and 20-HETE (160). It is well-established that MCs can release both prostaglandins and leukotrienes, whereas literature so far does not provide direct evidence of their ability to produce cytochrome P450 pathway derivatives. Interestingly, miR155, a well-known oncogenic miRNA found to be upregulated in several tumor types (180), can positively regulate FCεRI-mediated expression of COX enzymes in MCs (181).

Prostaglandins can exert pleiotropic functions in the TME (**Figure 3C**), depending on tumor type and on which type of cell (tumor or stromal) is targeted (159, 160). PGE_2_ can directly promote tumor cell proliferation, invasion, and metastasis (182, 183). It is endowed also with immunosuppressive effects, by promoting differentiation and tumor infiltration of MDSCs (184), facilitating M2 polarization (185) and upregulation of IL-1β and PD-L1 (14) in macrophages, inhibiting maturation and activity of DCs (186), and restraining effector functions of NK (187, 188) and T cells (189, 190). In line with this evidence, it has recently been shown that PGE_2_ and IL-1β produced by human PBMC-derived primary MCs can skew Th17 polarization, concomitantly restraining Treg suppressive functions, *in vitro* (191). However, the same function has been previously attributed to MC production of IL-6 triggered by OX40L-OX40 interaction, in a mouse model of autoimmune encephalomyelitis (139).

So far, literature has not directly shown that MCs produce PGE_2_ in the TME; nevertheless, evidence of PGE_2_ production by MCs in other contexts might support this hypothesis (192, 193). Yet, MCs are one of the main sources of PGD_2_ (155), with documented production also in cancer settings (194, 195). Notably, unlike PGE_2_, PGD_2_ signaling can restrain proliferation, migration, and survival in tumor cells (**Figure 3C**) (196, 197). Indeed, in the AOM/DSS mouse model, MC-derived PGD_2_ suppressed colitis and colitis-associated colon cancer (194). Furthermore, experiments using MC-deficient Kit^W-sh/W-sh^ mice, either reconstituted or not with MCs, demonstrated that MC-derived PGD_2_ exerts anti-angiogenic effects in the LLC lung cancer model (195). However, PGD_2_ shows several documented immunosuppressive functions in cancer, even if not directly associated with MCs so far. In acute promyelocytic leukemia (APL), it has been shown that PGD_2_ produced by tumor cells activates type 2 innate lymphoid cells (ILC2), which, in turn, foster M-MDSCs towards immunosuppression and dampening of the anti-tumor immune response (198). Experiments in mouse models and patients with melanoma demonstrated that PGD_2_ sustains an autocrine loop in TAMs, promoting their protumor functions. The same study also showed that macrophage-derived PGD_2_ inhibits CD8 T cell activation, contributing to the resistance to anti-PD1 immunocheckpoint therapy (199). Furthermore, in the B16F10 melanoma model, PGD_2_ was able to restrain IFNγ production, but not IL-4, by invariant NKT cells, reducing the protective effects of the iNKT ligand alpha-GalCer against experimental metastasis, *in vivo* (200). Finally, experiments in different mouse tumor models (melanoma, lung, and colon cancer) showed that PGD_2_ produced by T follicular helper (Tfh) cells can recruit Th2 cells within the tumor and stimulate their production of IL-4, thus promoting tumor growth (201). Notably, MC-derived PGD_2_ was demonstrated to be able to stimulate production of immunosuppressive cytokines (IL-4, IL-5, IL-13) by Th2 lymphocytes, independently of T cell receptor activation and co-stimulation, in a human cell culture system (202). Therefore, we can speculate that MC-derived PGD_2_ could also exert tumor-promoting functions by fostering an immunosuppressive TME. The balance between anti- and pro-tumor functions might be dictated by several factors, including tumor type and TME composition.

Similar to prostaglandins, leukotrienes, and in particular LTB_4,_ have multiple effects in the TME (159), mainly related to chemotactic activity on immune cell subsets with different pro- or anti- tumor activity. Indeed, studies in mouse models showed that LTB_4_ is implicated in the recruitment of M2 macrophages in lung cancer (203), and of T and NK lymphocytes in cervical cancer (204) and melanoma (205). Other models showed the opposite: depletion of the LTB_4_ receptor BLT1 was associated to reduced MDSC infiltration, increased DC recruitment and activity, and efficient antitumor immune response in a leukemia model (206). LTB_4_ has also been shown to mediate polarization of regulatory B cells (Breg) in breast cancer (207). Evidence of a direct role of MC-derived LTB_4_ in the TME has also been provided (**Figure 3C**). In lung cancer, LTB_4_ produced by MCs and macrophages can stimulate the production of crystalline silica, which, in turn, fosters lung chronic inflammation (silicosis) that can ultimately lead to lung cancer. The depletion of BLT1 was associated to reduced lung inflammation and tumor growth in spontaneous and subcutaneous models of lung cancer (208). Furthermore, it has been demonstrated that in the APC^Min^/^+^ model of colon cancer MCs produce LTB_4_ to recruit CD8 T cells, towards the generation of an effective antitumor immune response (209). Collectively, this information highlights that, as already shown for other mediators, the production of leukotrienes by MCs may result in pro- or anti-tumor effects, depending on tumor type and on the peculiar TME.

Other lipid mediators produced by MCs, with a relevant role in the allergic response, include PAF and S1P (162). Both molecules also display pleiotropic functions in the TME. PAF can foster immunosuppression by promoting the accumulation of PMN-MDSCs (210) and M2 macrophages (211). Furthermore, PAF has been shown to promote tumor growth (212), angiogenesis, metastasis (213, 214), and chemotherapy response (215). Similarly, S1P can promote cancer progression by supporting tumor growth, immune evasion, angiogenesis, metastasis, and therapy resistance (216, 217). Although, in this situation as well, a clear demonstration of MC production of PAF and S1P in the TME is lacking, the available findings let us suppose once again that MCs could be a relevant source of these lipid mediators in the TME, a hypothesis that warrants further investigation.

#### 6.2.4 Hypoxia

MCs are not primary drivers of hypoxia. Nevertheless, as outlined in the previous sections, they can survive in hypoxic environments and respond to them by generating pro-angiogenic and pro-inflammatory mediators, including VEGF (115), TNFα and IL-6 (116), which can, in turn, exacerbate hypoxia-associated features in the TME (**Figure 3D**).

As shown in this chapter, only a small number of studies have investigated the effect of MC-derived metabolites *in vivo* in the context of cancer (**Table 1**). In these works, PGD_2_ and LTB_4_ produced by MCs were able to limit tumor growth and foster T cell recruitment. Yet, other pieces of literature that we discussed above, allow us to speculate that other MC-derived metabolites (including histamine, lactate, PGE2, and PGD2 itself) could promote tumor cell growth and immunosuppression in the TME. The ability of MCs to survive in hypoxic conditions, where their functions are amplified, may further support their pro-tumorigenic role. All these unexplored aspects need to be clarified in future studies to better elucidate the metabolic basis of MC functions in the TME.

## 7 Discussion, conclusions, and perspectives

In consideration of the existing literature, it is possible to formulate several definitive observations. Primarily, there is an evident paucity of studies focused on metabolic changes of MCs within a tumor context. In this review, we have discussed several works that addressed either the effect of different stimuli on MC metabolism or the impact of MC-derived metabolites on target cells in several non-cancer pathological contexts (allergies, acute or chronic inflammation, etc.). From these findings, we have inferred potential similarities that could be applied to the tumor setting. We have also described effects exerted by metabolites produced by other cell types in the TME, which MCs can also produce, to hypothesize potential unexplored metabolism-associated MC functions in cancer. Conversely, only a few works directly dissected MC sensing and perturbation of the metabolite composition in the TME. Moreover, many of the studies cited in this review rely on *in vitro* experiments, which investigated the impact of a single metabolite on a specific type of MC. Therefore, these works do not consider the substantial variability of cell accomplices, molecules, and mediators that is characteristic of the TME *in vivo*, and do not take into account the considerable heterogeneity that is observed among MCs in different tissues, nor the divergent responses that MCs can exhibit depending on the type and timing of the received stimulation. Furthermore, as previously mentioned, metabolic heterogeneity within the TME is determined by several local and systemic factors, including tumor type, TME composition, microbiota, diet, and nutritional status of the patient. All these variables could further impact MC metabolism and function. Nevertheless, it is important to note that all the studies discussed here have allowed us to draw important inferences, which, however, are awaiting further confirmation in future works.

To properly investigate how the metabolic-related functions of MCs can shape tumor and stromal cells, and vice versa, how the TME can affect MC metabolism and function, an integration of several experimental approaches should be implemented.

*In vivo* experiments will have to compare injection of tumor cells in MC-proficient and deficient mice (e.g. Kit^w-sh/w-sh^, MCPT4 knock-out, Cpa3-Cre, and MCPT5-Cre mice (218–221)). The latter could be adoptively transferred with MCs specifically lacking genes of interest related to metabolic pathways under investigation (e.g. BMMCs coming from MCPT4 knock-out mice, where lactate efflux is impaired). Similarly, tumor cells could be rendered knock out for genes of interest. These experiments could have multiple readouts. To best dissect their metabolic function, MCs isolated *ex vivo* (via FACS or magnetic beads) from tumors could be analyzed by Seahorse. Alternatively, experiments could involve *in vivo* administration of fluorescent dyes (e.g., BODIPY or MitoTracker, TMRM) to assess lipid uptake and mitochondrial activity of MCs and other cells within the TME by flow cytometry. Single-cell RNA sequencing analyses will allow a comprehensive analysis of metabolic-related pathways of tumor, MCs, and other TME cells. Furthermore, more sophisticated technologies such as spatial metabolomics [e.g. MALDI Imaging (222)] and single-cell metabolomics (223, 224) could be applied for the evaluation of lipids and other metabolites in tumor tissues.

Finally, we think that these approaches could be jointly applied and complemented by *ad hoc*-designed *in vitro* experiments, aimed at better dissecting molecular and metabolic interplays, to strengthen the results. The ultimate goal will be to validate some of the findings obtained in the preclinical models in tumor specimens collected from cancer patients.

Another important aspect that warrants discussion and further investigation is the possibility of targeting MC metabolism for cancer therapy. Indeed, given the importance of metabolism for tumor growth and regulation of TME functions, several therapeutic strategies targeting metabolism have been proposed for cancer. These approaches, either nutritional or pharmacological, have been extensively reviewed elsewhere (61, 62, 64, 225). Interestingly, numerous pharmaceutical agents targeting cell metabolism, currently employed in the treatment of non-oncological diseases, are alongside demonstrating therapeutic potential in the context of cancer. Given the evidence reported in this review, it can be speculated that some of these pharmacologic or dietary interventions could impact MC metabolism, likely influencing their activity in the TME. It will be worth investigating, also utilizing the aforementioned experimental approaches and techniques, whether the outcome of these interventions will result in enhanced pro- or anti-tumor effects of MCs, also in relation to the peculiar tumor setting.

For instance, metformin, a common anti-diabetic drug, seems to have potential in the cancer context as well (226). It should be noted that metformin has been shown to modify MC activity, restraining both IgE- and AHR- mediated responses (227). Similarly, non-steroidal anti-inflammatory drugs inhibiting the activity of COX enzymes, such as aspirin and celecoxib, have been shown to be cancer-preventive in several clinical trials (228, 229). Yet, as these drugs can impact the production of prostaglandins, it can be speculated that they might affect MC function in the TME, by impacting on PGE_2_-mediated MC activation and recruitment, or by inhibiting PGD_2_ production by MCs. In support to this hypothesis, it has been shown that aspirin can reduce the otherwise abnormal PGD_2_ levels in a small cohort of patients with systemic mastocytosis (230). Nevertheless, MCs are established mediators in the pathophysiology of aspirin-exacerbated respiratory disease (AERD (231);), a systemic inflammatory condition induced by overreaction to aspirin or other COX inhibitors, and characterized by dysregulated arachidonic acid metabolism leading to reduced PGE_2_ and increased leukotriene production. Therefore, the effects of aspirin on tumor-infiltrating MCs could be context-dependent. Similar considerations could be drawn for the leukotriene receptor antagonist montelukast, which is commonly used in asthma treatment and can inhibit MC recruitment (232, 233), and has also shown promising anti-cancer effects in preclinical studies in several tumor types (234, 235). In conclusion, despite the growing interest in tumor metabolism, the metabolic rewiring of MCs within the TME remains relatively underexplored compared to other immune cell types. A deeper understanding of MC-specific metabolic networks in the TME is needed, not only to elucidate their functional modulation in response to current metabolic interventions (pharmacologic or dietary), but also to explore their potential as therapeutic targets in the context of cancer immunometabolism.

## Glossary

Glycolysis: pathway that occurs in the cytosol. It allows to produce two pyruvate molecules, two ATP molecules and two NADH molecules from one glucose molecule
TCA (tricarboxylic acid) cycle: pathway that takes place in the mitochondrial matrix, where it processes acetate derived from carbohydrates, proteins, and fats to generate key metabolic intermediates, including ATP, NADH, and FADH_2_
OXPHOS (oxidative phosphorylation): this process, occurring in the inner mitochondrial membrane, involves the transfer of electrons from NADH and FADH_2_
to molecular oxygen via the electron transport chain (ETC): generating an electrochemical gradient that drives ATP synthesis
ECAR (extracellular acidification rate): parameter used to estimate glycolysis levels
OCR (oxygen consumption rate): parameter used to estimate mitochondrial respiration levels
SCFA (short chain fatty acids): acetate, propionate and butyrate are the main SCFA that are metabolic products of gut bacteria on indigestible fibers
2-DG (2-deoxy-d-glucose): glucose analog that interferes with glycolysis, able to enter the cells through glucose transporters
